# Effects of Various Flavonoids on the *α*-Synuclein Fibrillation Process

**DOI:** 10.4061/2010/650794

**Published:** 2010-01-28

**Authors:** Xiaoyun Meng, Larissa A. Munishkina, Anthony L. Fink, Vladimir N. Uversky

**Affiliations:** ^1^Department of Chemistry, University of California, Santa Cruz, CA 95064, USA; ^2^Center for Computational Biology and Bioinformatics, Department of Biochemistry and Molecular Biology, Institute for Intrinsically Disordered Protein Research, Indiana University School of Medicine, 410 W. 10th Street, HS 5009, Indianapolis, IN 46202, USA; ^3^Institute for Biological Instrumentation, Russian Academy of Sciences, Pushchino, Moscow 142290, Russia

## Abstract

*α*-Synuclein aggregation and fibrillation are closely associated with the formation of Lewy bodies in neurons and are implicated in the causative pathogenesis of Parkinson's disease and other synucleinopathies. Currently, there is no approved therapeutic agent directed toward preventing the protein aggregation, which has been recently shown to have a key role in the cytotoxic nature of amyloidogenic proteins. Flavonoids, known as plant pigments, belong to a broad family of polyphenolic compounds. Over 4,000 flavonoids have been identified from various plants and foodstuffs derived from plants and have been demonstrated as potential neuroprotective agents. In this study 48 flavonoids belonging to several classes with structures differing in the position of double bonds and ring substituents were tested for their ability to inhibit the fibrillation of *α*-synuclein in vitro. A variety of flavonoids inhibited *α*-synuclein fibrillation, and most of the strong inhibitory flavonoids were also found to disaggregate preformed fibrils.

## 1. Introduction

Parkinson's disease (PD) is a chronic progressive disease, characterized clinically by resting tremor, bradykinesia (slowness in initiating movements), and rigidity. As only a small number of cases are diagnosed before the age of 50, PD is typically considered an aging disease, with a prevalence of approximately 2% after the age of 65 [1]. The cause of this neurodegenerative disease is still mysterious, although considerable evidence suggests a multifactorial etiology involving genetic susceptibility and environmental factors. 

In PD, neuronal death is localized to dopaminergic neurons in the substantia nigra region of the brain stem and precedes appearance of symptoms. It is believed that ca. 70% of neurons may have died by the time symptoms become apparent [2]. Diagnosis can only be definitively confirmed post mortem, by histopathological examination for the pigmented neuron loss and the presence of Lewy bodies (LBs) and Lewy neuritis (LNs) [3]. LBs are spherical protein inclusions found in the cytoplasm of surviving nigral neurons consisting of a dense core surrounded by a halo of radiating fibrils of *α*-synuclein, the major component of LBs [4]. A variety of other proteins have also been identified in LBs [5, 6]. However, both the mechanism underlying the formation of LBs and their pathogenic relevance are still unclear [7, 8].

Substantial evidence suggests that the aggregation of *α*-synuclein is a critical step in the etiology of PD [9–11]. The following observations are among the most compelling for the involvement of *α*-synuclein and its aggregation in PD: (i) fibrils of *α*-synuclein are observed in LBs and LNs, the characteristic hallmarks of the PD pathology [4, 12, 13]; (ii) three missense mutations, A53T, A30P, and E46K in *α*-synuclein, lead to familial early onset Parkinson's disease [14–16]; A53T and A30P were known to increase the aggregation propensity of *α*-synuclein [17, 18]; (iii) genetic studies have shown that several cases of familial early onset PD are caused by overexpression of *α*-synuclein due to duplication or triplication of the *α*-synuclein gene locus (*SNCA*) [19, 20]. 

Several possible mechanisms for the observed cytotoxicity of amyloidogenic assemblies have been suggested. One mechanism suggests a direct effect of amyloidogenic assemblies on cell membrane structure and integrity, either through the pore formation [21–26], or via the destabilization of amyloidogenic bundles [27, 28]. 

Currently, no preventive therapy is available for PD. It is believed that compounds that slow and/or prevent aggregation and fibrillation of *α*-synuclein could represent potential drug leads for PD prevention. Therefore, many of the latest therapeutic strategies are aimed at inhibiting fibril formation and at promoting fibril clearance. They include the use of antibodies, synthetic peptides, molecular chaperones, and chemical compounds. In the past few years small organic molecules, especially certain polyphenolic compounds, have been extensively tested for their ability to inhibit fibril formation in vitro, particularly in relation to A*β* deposition [29], the formation of protease-resistant forms of the prion protein [30], the aggregation of huntingtin [31], and the heparin-induced formation of tau filaments [32, 33]. An analysis of these reports shows that certain polyphenols dramatically inhibit cell death in the amyloidogenic cytotoxicity assays. 

Less is known about effects of polyphenols and other small organic molecules on *α*-synuclein fibril formation. Only the antituberculosis drug rifampicin and Chinese herbal drug baicalein from *Scutellaria baicalensis* have been analyzed and shown to inhibit *α*-synuclein fibrillation [36–38]. 

**Figure 1:**
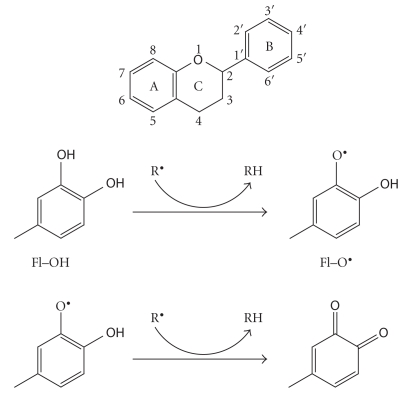
(a) Basic structure of a flavonoid, a C15-skeleton showing the A, B, and C ring, reproduced from [34]. (b) Scavenging of ROS (R^•^  ) by flavonoids, reproduced from [35].

Flavonoids have been known as plant pigments for over a century and belong to a broad class of polyphenolic compounds. Over 4,000 flavonoids have been identified from various plants [39] and foodstuffs derived from plants, forming substantial constituents of the human diet. The intake of flavonoids is in the range of 50–800 mg/day, depending on the consumption of vegetables and fruits and of specific beverages, such as red wine, tea, and unfiltered beer [40]. Most of the flavonoids are very potent antioxidants, which has often been associated with their health-related effect. Due to their low redox potentials (0.23 < E7 < 0.75 V) [41], flavonoids (Fl–OH) are thermodynamically able to reduce highly oxidizing free radicals with redox potentials in the range 2.13–1.0 V [42], by hydrogen atom donation:

(1) $$\text{Fl–OH}+{\text{R}}^{•}\to \text{Fl–}{\text{O}}^{•}+\text{RH}$$

where R^•^ represents superoxide anion, peroxyl, alkoxyl, and hydroxyl radicals [43–45]. The aroxyl radical (Fl–O^•^) may react with a second radical, acquiring a stable quinone structure (Figure 1(b)). Scavenging of free radicals plays a considerable role in the antioxidant activity of flavonoid compounds [46]. In recent years, flavonoids, being potent free radical scavengers, have attracted a tremendous interest as possible therapeutics against free radical-mediated diseases associated with the oxidative stress [47]. 

Oxidative stress is an imbalanced state where excessive quantities of reactive oxygen species (ROS), such as superoxide radical anion, hydrogen peroxide, and hydroxyl radical, are present at levels higher than required for normal cell function and overwhelm endogenous antioxidant capacity and repair [48, 49]. Oxidative stress has been linked to cancer, aging, atherosclerosis, ischemic injury, inflammation, and neurodegenerative diseases such as Parkinson's and Alzheimer's diseases [50–53]. 

As recently reviewed by Pietta [34], several attempts have been made by numerous authors to establish the relationship between flavonoid structure and their radical-scavenging activity [54–57]. In general, the radical-scavenging activities of flavonoids depend on the molecular structure and the substitution pattern of hydroxyl groups, that is, on the availability of phenolic hydrogens and on the possibility of stabilization of the resulting phenoxyl radicals via hydrogen bonding or by expanded electron delocalization [58–60]. Besides the radical scavenging activity, the ability of flavonoids to chelate (bind) metal ions also contributes to their antioxidant activity in vitro. The proposed binding sites for trace metals to flavonoids are basically consistent with the structural requirements for effective radical scavenging, suggesting that metal chelation and antioxidant activity are intimately linked.

Oxidative stress, transition metal accumulation, and inflammation appear to play a role in the pathology of several neurodegenerative diseases, including Parkinson's disease. Accumulated evidence has suggested that dietary flavonoids could be potential neuroprotective agents. For example, the consumption of flavonoid-rich blueberries or strawberries can reverse cognitive and motor behavior deficits in rats [62], and intake of antioxidant flavonoids is associated with a lower incidence of dementia [63]. EGCG, a flavonoid from green tea, has been shown to be neuroprotective in an MPTP-induced animal model of dopaminergic degeneration by Levites et al. [64]. The data from Griffioen et al. also demonstrated that several flavonoids counteract the cytotoxic properties of *α*-synuclein aggregates directly in a cellular context [65].

The properties of flavonoids such as their abundance in plants and human diet, variety in composition and structure, and antioxidative and neuroprotective biological activities make these compounds very attractive targets while screening small organic molecules that can interact with *α*-synuclein protein and inhibit its aggregation. These natural chemical compounds can be potentially used as therapeutic drugs in preventing of neurodegeneration in Parkinson's disease. Therefore, we analyzed a flavonoid library containing 48 compounds from several classes for their ability to inhibit *α*-synuclein fibril formation in vitro. Many flavonoids were shown to inhibit *α*-synuclein fibrillation and aggregation. Importantly, the majority of the strong inhibitors also disaggregate the preformed fibrils.

## 2. Materials and Methods

### 2.1. Materials

All flavonoids were obtained from INDOFINE chemical Co., Inc. Thioflavin T (ThT) was obtained from Sigma-Aldrich. All other chemicals were analytical grade and obtained from Fisher chemicals or VWR scientific.

### 2.2. Expression and Purification of *α*-Synuclein

Wild type (WT) human recombinant *α*-synuclein-containing plasmid *pRK172* (obtained from R. Jakes and M. Goedert) was transfected into *Escherichia coli* BL21 (DE3) cells. Protein was induced and purified as described previously [66]. Protein concentration was determined spectrophotometrically.

### 2.3. Aggregation/Fibrillation Studies and ThT Assays

Lyophylized *α*-synuclein was prepared for use by dissolution at high pH (approximately 10.5) in dilute NaOH for 5 to 10 minutes and adjusted to pH 7.4 prior to centrifugation at 95,000 rpm with a Beckman Airfuge ultracentrifuge to remove any aggregated material. A 5-fold dilution with buffer (see text for specific conditions used) was followed with adjustment to the desired pH. Flavonoids were dissolved in DMSO to make a stock solution at a concentration of 5, 10, or 20 mm and to make a final solution of protein and flavonoid in 1% DMSO unless indicated otherwise. All of the protein final solutions consisted of PBS with 0.02% NaN_3_ added to avoid bacteria growth. 

For plate assays, the protein solution (0.5 mg/mL or 35 *μ*M) was mixed with flavonoids to give a final concentration of 50 *μ*M. Three replicates of each sample were prepared in a 96-well plate with a volume of 120 *μ*L per well and a 3 mm Teflon bead to provide agitation (120 rpm, 20 mm diameter). The assays were performed in a fluorescent plate reader (Labsystems Fluoroskan Ascent CF). Measurements were taken at 30-minute intervals with excitation at 444 nm and emission monitored at 485 nm. 

For manual assays, the protein solution (1 mg/mL or 70 *μ*M) was mixed with flavonoids to give a final concentration of 100 *μ*M. Protein samples were stirred at 37°C with a mini-Teflon stir bar. Aliquots of 5 or 10 *μ*L were removed from the incubated solution and added to 1 mL of 10 *μ*M ThT solutions in 20 mm phosphate buffer (pH 7.4) as a function of time to monitor the fibrillation kinetics. ThT fluorescence was recorded at 482 nm with excitation at 450 nm and slits of 5 nm for both excitation and emission using a FluoroMax-3 spectrofluorometer (Jobin Yvon Horiba). 

### 2.4. Electron Microscopy Measurements

Transmission electron microscopy (TEM) was used to estimate the size and structural morphology of *α*-synuclein. Aliquots of 5 *μ*L sample were deposited on Formvar-coated 300 mesh copper grids (Ted Pella) and incubated for 5 to 10 minutes. Salts were washed out with distilled water, and samples were dried, negatively stained with 1% (w/v) uranyl acetate, and visualized on a JEOL JEM-100B transmission electron microscopy operated at 80 kV. Typical magnifications ranged from ×75,000 to 300,000. The grids were thoroughly examined to obtain an overall evaluation of the samples. Images were produced with Gattan Digital Micrograph software.

### 2.5. Electrophoresis

Sodium Dodecyl Sulfate Polyacrylamide Gel Electrophoresis (SDS-PAGE) was carried out on an Amersham Phast System Separation and Control Unit with PhastGel Gradient 8-25 polyacrylamide gels. SDS buffer (0.20 M tricine, 0.20 M Tris, 0.55% SDS, pH 8.1) strips (Amersham) were used. Gel electrophoresis samples were prepared by the addition of 3 parts of protein solution to 1 part SDS buffer (0.25 M Tris, 8% SDS, 60% glycerol, 0.08% bromophenol blue, pH 6.8) and boiled for 3–5 minutes. The 10–225 kDa protein markers were purchased from USB Corporation (Cleveland, Ohio, USA).

Isoelectric Focusing (IEF) Polyacrylamide Gel- IEF-Polyacrylamide Gel (4–6.5) was carried out on an Amersham Phast System Separation and Control Unit with IEF polyacrylamide gels. PI standards contain proteins as follows: Human carbonic anhydrase B: 6.55; Bovine carbonic anhydrase B: 5.85; *β*-Lactoglobulin A: 5.20; Soybean trypsin inhibitor: 4.55; Glucose oxidase mannitol: 4.15; Methyl red (dye): 3.75; Amyloglucosidase: 3.50; Pepsinogen: 2.80.

### 2.6. Chromatography: Size Exclusion (SEC) HPLC Measurements

An aliquot of each sample was removed from the *α*-synuclein incubation at various times, and the insoluble material was removed by centrifugation for 20 minutes at 14,000 rpm. Sample volumes of 40 *μ*L of supernatant were eluted from a TSK-GEL G4000SWXL size exclusion column (7.8 mm inner diameter × 30 cm) in 20 mm phosphate buffer, pH 7.0, and 100 mm Na_2_SO_4_ using a Waters 2695 separations module with a Waters 996 photodiode array detector. The HPLC system was controlled, and data were collected and analyzed by Millennium software. The column was eluted at a flow rate of 0.5 mL/min, and the absorbance of the mobile phase was monitored over the wavelength range from 220 to 450 nm with a bandwidth of 1.2 nm. The retention times were calibrated with the following protein molecular mass standards: ribonuclease A (13.6 kDa), chymotrypsinogen A (25 kDa), ovalbumin (43 kDa), albumin (bovine serum) (67 kDa), and aldolase (158 kDa). The void volume was determined with blue dextran 2000 (~2000 kDa).

### 2.7. Electrospray Ionization Mass Spectrometry (ESI-MS)

Samples for mass spectrometry analysis were desalted with C8 reverse phase column and eluted out with 80% acetonitrile adjusted with TFA to a final pH at 2.0. Injection was carried out via a Harvard Apparatus (Holliston, MA) syringe pump at a flow rate of 20 *μ*L/min. Mass spectra were obtained using Micromass ZMD electrospray mass spectrometer operating in positive ionization mode. The source temperature was set to 80°C, and the capillary voltage was 3.25 kV. Protein molecular weight was determined from m/z by Masslynx software.

## 3. Results and Discussion

### 3.1. Effects of Various Flavonoids on the Fibrillation of *α*-Synuclein

#### 3.1.1. Fibrillation Kinetics of WT *α*-Synuclein in the Presence of Various Flavonoids

In this study, 48 flavonoids belonging to several classes with structures differing in ring substituents and in the nature/extent of alkylation have been tested for their ability to inhibit the fibrillation of *α*-synuclein in vitro. These flavonoids include 18 flavones, 12 flavonols, 4 flavanones, 6 isoflavones, 2 dihydroflavonols, 3 catechins, and 3 anthraquinones. Flavonols and flavones accounted for the majority of the study because of their wide distribution in foods. Table S1 (see Table S1 in Supplementary Materials available online at doi:10.1061/2010/650794.) lists the name, synonym, and structure of the flavonoid from each class. The basic flavonoid structure is the flavan core, which consists of a 15 carbon skeleton (C6-C3-C6), arranged in three rings labeled as A, B, and C in Figure 1(a) [34]. The various classes of flavonoids differ in the level of oxidation and pattern of substitution of the C ring, including flavonols (quercetin and kaempherol), flavanols (the catechins), flavones (apigenin), and isoflavones (genistein). Individual compounds within a class differ in the pattern of substitution of the A and B rings. 

**Figure 2:**
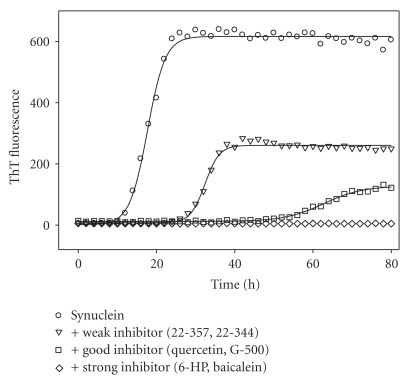
Inhibitory effects of flavonoids on the kinetics of *α*-synuclein fibrillation. Comparison is made between strong inhibitors (+++), such as 6-HP and Baicalein, good inhibitors (++), such as quercetin and G-500, and weak inhibitors (+), such as 22-357 and 22-344.

Figure 2 represents kinetic profiles of *α*-synuclein fibril formation in the absence (circles) or presence of different flavanoids, as monitored by changes in the ThT fluorescence. Different flavonoids affected the *α*-synuclein fibrillation to a different extend. Some flavonoids, such as 22-357 and 22-344 (Figure 2, inverted triangles), slightly inhibited the fibrillation of *α*-synuclein, increasing the lag time 2- to 3-fold in comparison with that of the control, and were classified as weak (+) inhibitors. Other flavonoids, such as quercetin and G-500 (Figure 2, squares), significantly inhibited *α*-synuclein fibrillation, with the lag time 4- to 5-fold longer than that of the control, and were classified as good (++) inhibitors. 

In the presence of 6-HP (Figure 2, diamonds), *α*-synuclein fibrillation was completely inhibited, as indicated by the negligible changes in the ThT fluorescence over the time course. More importantly, no detectable *α*-synuclein fibrillation was observed for about 3 days or longer. The inhibitory effect of 6-HP on *α*-synuclein fibrillation was as good as that from baicalein (Figure 2), which was reported earlier by Zhu et al. [36]. Besides 6-HP, several other new flavonoids from different classes including eriodictoyl, 22-324, myricetin, EGCG, T-415, 22-340/tricetin, and 22-341, also exhibited strong inhibitory effects on *α*-synuclein fibrillation. Flavonoids that completely inhibited the fibril formation of *α*-synuclein under the current experimental conditions were classified as strong inhibitors (designated as +++**)**. The inhibitory activities of each flavonoid analyzed in this study are listed in Table S1 (see Supplementary Table S1). 

**Figure 3:**
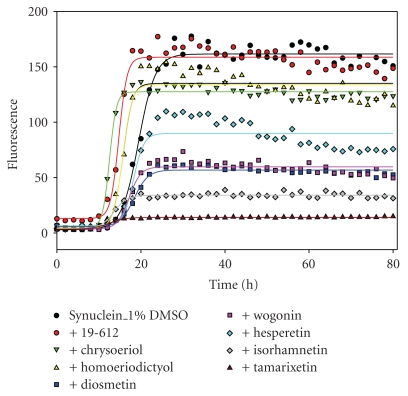
The kinetics of *α*-synuclein fibrillation in the presence of noninhibitory flavonoids by ThT plate reader assays. Each curve was obtained by fitting the data with Sigma Plot, using the equation from [61].

In general, the intensity of ThT fluorescence was proportional to the amount of fibril forms. For instance, in the presence of the inhibitory flavonoids, the fluorescence intensity at the end of reaction was in the decreasing order: control (no flavanoid) > 22-257 > quercetin > 6-HP. EM analysis revealed that the detectable amount of fibrils formed at the end of the reaction was decreasing in the same order. In the plate assays, however, most of the tested compounds gave artificially low ThT signal. For example, in the presence of some flavonoids, such as wogonin, diosmetin, and tamarixetin (Figure 3), the ThT fluorescence intensity was much lower than the control, whereas the kinetics of *α*-synuclein fibrillation still exhibited a typical sigmoidal shape, with the lag time similar to the control. Since the amount of fibrils formed at the end of reaction was found to be similar to that of the control, as indicated by the EM imaging, the lower ThT fluorescence intensity for samples containing these flavonoids suggested that flavanoids either quenched the ThT fluorescence or competed with ThT at the binding site on the protein fibrils. Therefore, no matter how much the flavonoids diminished the ThT fluorescence, as long as they did not prolong the lag time of *α*-synuclein fibrillation, such as 19-612, chrysoeriol, wogonin, and tamarixetin, and they were all classified as noninhibitors (designated as **−**). 

**Figure 4:**
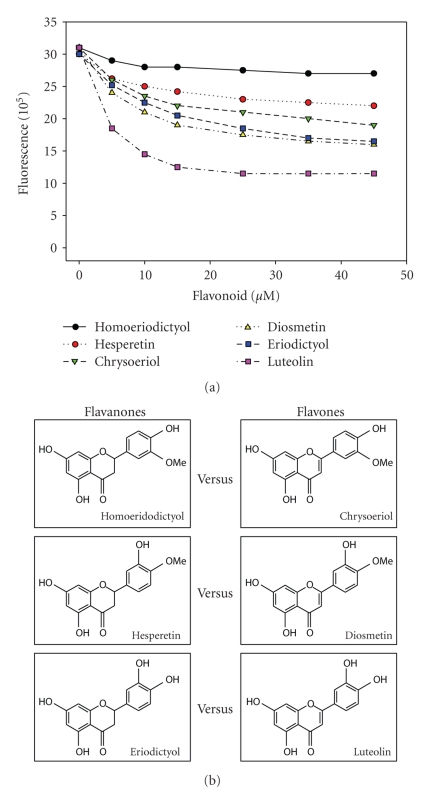
Titration of existing fibril-ThT complex by flavonoids. Structures of the flavonoids are listed on the right side. ThT concentration: 10 *μ*M. Cuvette volume: 1 mL. With excitation at 450 nm, fluorescence emission at 482 nm was measured upon the addition of flavonoid.

The quenching/competition was verified by titrations of fibril-ThT complex with various flavonoids. The increasing concentrations of flavonoids from different classes were added to a certain amount of existing fibrils in the presence of 10 *μ*M ThT solution. The intensity of ThT fluorescence in the presence of fibrils decreased as a function of flavanoid added and approached a certain saturation level when the concentration of the flavonoid was around 50 *μ*M (Figure 4). The decrement degree was dependent on the number and the arrangement of hydroxyl groups for flavonoids from the same class (e.g., flavanones: homoeriodictyol, hesperetin, and eriodicyol) and on the level of saturation of Ring C for flavonoids from different classes but with comparable structures (e.g., flavanones with 2, 3 double bond in Ring C compared with the corresponding flavones). 

Figure S2 (see Supplementary Figure S2) shows instant changes in the ThT fluorescence intensity measured in the presence of *α*-synuclein fibrils and 50 *μ*M flavonoids. The addition of several flavonoids, such as 6-HP, myricetin, G-500, and emodin, resulted in almost 20-fold decrease in the ThT fluorescence intensity in comparison with the control signal.

**Figure 5:**
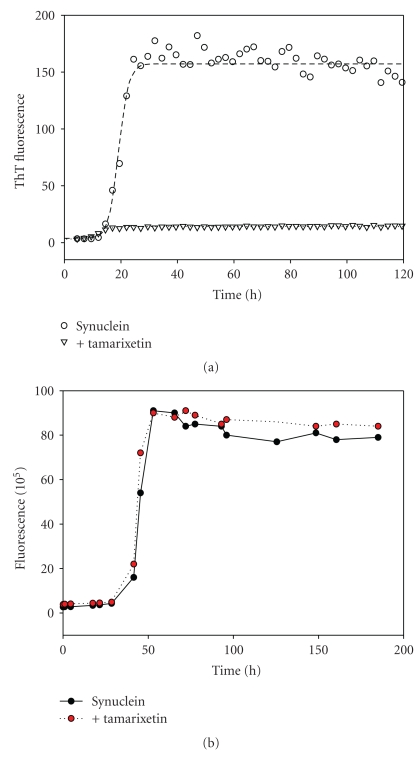
The effect of tamarixetin on the ThT fluorescence of *α*-synuclein fibrillation by two ThT assays: the plate reader assay (a) and the manual assay (b).

To avoid interference of flavonoids on ThT fluorescence, manual ThT assays were employed. The final ratio of flavonoid to ThT in the manual assay was lower (about 1 : 10) compared with that in the plate assay; therefore the quenching by flavonoids was almost completely eliminated.  Figure 5 compares the results of the plate assay and the manual assay for the fibrillation of *α*-synuclein in the presence of one of the non-inhibitory flavonoids, tamarixetin. In the plate assay, ThT fluorescence intensity in the presence of 50 *μ*M tamarixetin was much lower (~10-fold) than that in the absence of the flavonoid, whereas in the manual assay, the addition of 50 *μ*M tamarixetin did not affect ThT fluorescence intensity (Figure 5). Similar results were obtained when manual ThT assays were performed with other non-inhibitory flavonoids (data not shown).

All potential inhibitory flavonoids identified in high-throughput plate-reader assays were tested in subsequent manual assays. In addition, the presence of fibrils was evaluated by EM and quantitatively estimated from SDS PAGE of supernatant fractions (described below).

### 3.2. Analysis of *α*-Synuclein Species Formed in the Presence of Flavonoids

**Figure 6:**
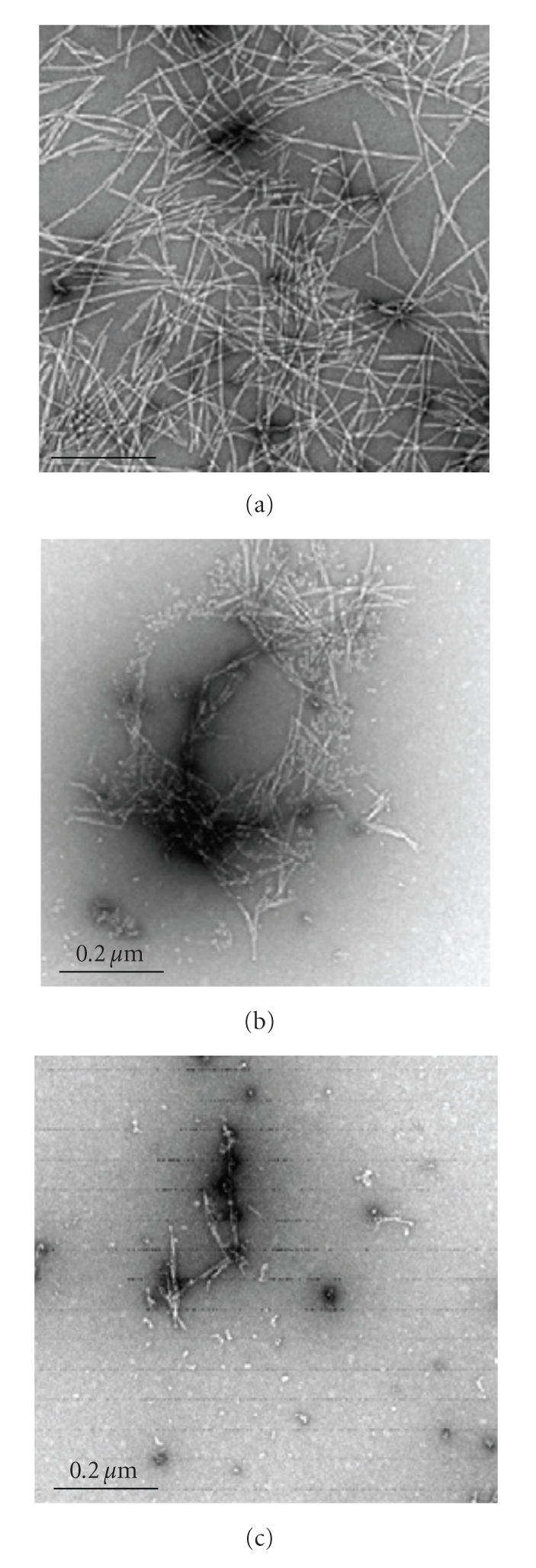
TEM images of *α*-synuclein samples after 3 days of incubation in the absence (a) and presence of weak/good (b) and strong (c) inhibitors of *α*-synuclein fibrillation.

During storage or incubation, monomeric *α*-synuclein tends to self-associate into complexes of various degrees including dimers, trimers, oligomers of various size and shapes, protofibrils, and fibrils. Based on EM, DLS, SEC, and SDS-PAGE, the typical distribution of *α*-synuclein species after 3 days of incubation was 75%–85% of fibrils, 5%–10% of protofibrils, and 5%–20% of oligomeric species and amorphous aggregates. The non-inhibitory flavonoids did not affect the distribution and morphology of fibrils (Figure 6(a)). Typical fibrils were 10 ± 1 nm in width and up to about 2 *μ*m in length. Flavonoids classified as weak or good inhibitors decreased fibril amounts. Remained fibrils were shorter in length but similar in width (Figure 6(b)). When *α*-synuclein was incubated with strong inhibitory flavonoids only oligomers and a few protofibrils instead of fibrils were detected (Figure 6(c)). Flavonoid-induced protofibles were 5–10 times shorter in length and twice smaller in width compared with the typical *α*-synuclein fibrils. Flavonoid-induced oligomers appeared as round- or oval-shaped species with a 10–20 nm diameter. Their morphology was similar to the oligomeric species observed in the absence of flavonoids; however, the detailed structure and assembly could be different. Unfortunately, this is beyond the detection limits of AFM and EM. Inhibitory flavonoids greatly affected the distribution of aggregated *α*-synuclein species apparently by stabilizing monomeric and oligomeric species. 

**Figure 7:**
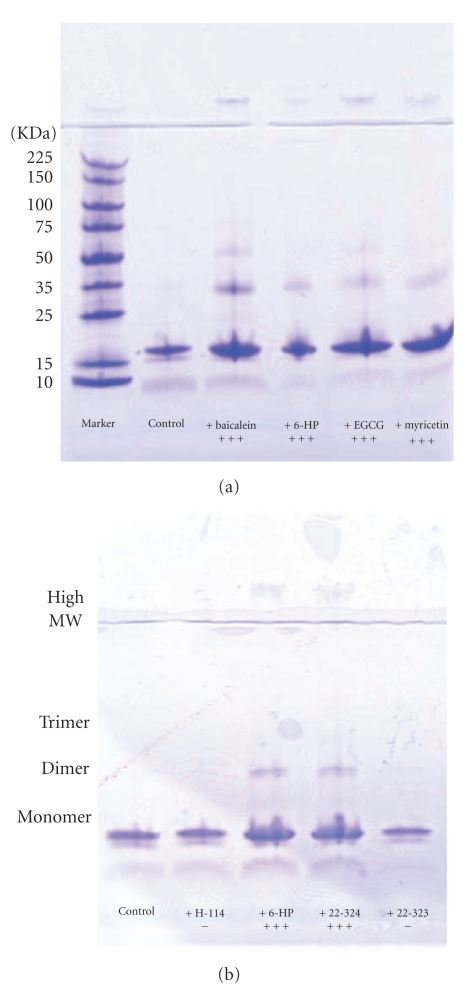
(a) SDS-PAGE from the supernatants of the incubated samples of *α*-synuclein in the absence and presence of the strong inhibitory flavonoids. (b) Comparison of the effect of strong inhibitory flavonoids (6-HP, 22-324) with noninhibitory flavonoids (H-114, 22-323) on formation of stabilized soluble species.

The distribution and stability of soluble species of *α*-synuclein were analyzed by SEC and SDS-PAGE. In addition, SDS PAGE was employed to quantitatively estimate the amount of protein in the supernatant and pellet fractions because Lowry and other spectrophotometric methods could not be used due to flavonoid interference with the absorbance.  Figure 7(a) shows SDS-PAGE of supernatant fractions of *α*-synuclein samples incubated in the absence or presence of the strong inhibitory flavonoids. In the control lane, a weak band of monomeric *α*-synuclein was observed indicating that most of the protein was insoluble. No dimers or oligomers were detected. In the presence of non-inhibitory flavonoids such as H-114 and 22-323, there was also a relatively weak band of monomeric *α*-synuclein in the gel (Figure 7(b)), similar to that of the control further confirming that these flavonoids did not affect *α*-synuclein fibrillation. On the other hand in the presence of strong inhibitory flavonoids such as 6-HP and 22-324, essentially all of the protein was present in the supernatant. The monomer band was much intense than that of the control. Importantly, higher molecular weight species were also detected on SDS gel. They correspond to dimers (35 kDa band), trimers (50 kDa band), and oligomers (a band on the top of the gel). The oligomeric species and dimer/trimers were resistant to treatment with SDS at 100°C for 20 minutes. They were also extremely stable in 8 M urea and 6 M GdmHCl (data not shown). The stability of these oligomers and dimers indicates that they are different from the similar species formed in the absence of flavonoids, which are unstable in the mentioned conditions. The extreme stability can be due to very strong noncovalent interactions and/or covalent binding.

**Figure 8:**
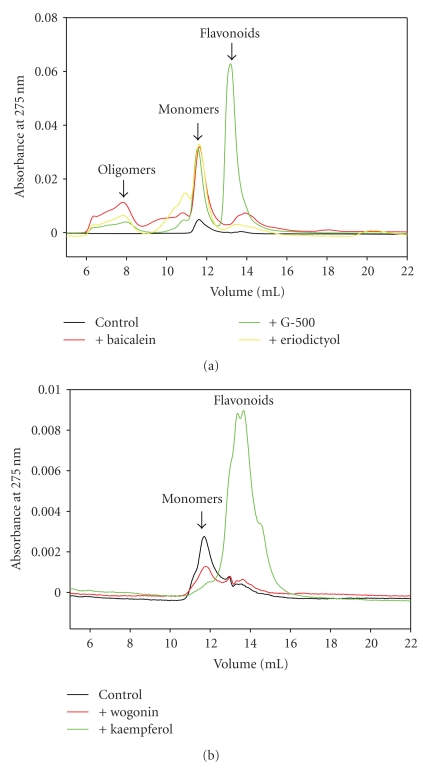
SEC-HPLC profiles of the supernatants of *α*-synuclein samples incubated in the absence and presence of various flavonoids: inhibitory flavonoids (a) and non-inhibitory flavonoids (b).

The soluble forms of *α*-synuclein stabilized by the flavonoids were further analyzed by size exclusion HPLC. The elution profile of *α*-synuclein control sample showed only one peak at 11.5 mL corresponding to a trace amount of monomers (Figure 8, black curves). These results were rather unexpected as EM, AFM, and DLS analyses revealed that oligomers constituted the majority of the supernatant fractions (data not shown). This contradiction can be explained by the instability of oligomers under HPLC condition and by their fast dissociation upon dilution during chromatographic process. On the contrary, SEC analysis revealed that soluble fractions of *α*-synuclein incubated with flavonoids contained significant amounts of oligomers. Oligomeric fractions of *α*-synuclein were obtained by running FPLC, which required much more protein for analysis and was used only for some flavonoid-protein samples (data not shown). The elution profiles of *α*-synuclein incubated in the presence of non-inhibitory flavonoids such as wogonin and kaempferol were similar to the control profile (Figure 8(b)). Peaks eluted after the monomer peak (13.5 and 14 mL) corresponded to polymerized forms of flavonoids that occurred during incubation [67]. The flavonoid nature of the peaks was experimentally confirmed by the same elution times and absorbance spectra of incubated flavonoid alone and by the absence of protein band on SDS gel for the concentrated fraction sample from SEC-HPLC (data not shown).

The elution profiles of *α*-synuclein incubated in the presence of inhibitory flavonoids had six major peaks. Besides the monomer and flavonoid peaks, peaks corresponding to dimer (11 mL), trimer (10.3 mL), and small (7.8 mL) and large oligomer (6.2 mL) were detected (Figure 8). Because of the flavonoid interference with the protein spectra, the protein nature of the peaks and the amount of protein eluted was analyzed by SDS-PAGE. The increased intensity of the monomer peak was mostly attributed to the elevated amount of monomers and partially to the absorbance of flavonoid tightly bound to the monomer. Absorbance intensity of dimer and oligomer peaks also partially corresponded to the flavonoid absorbance and indicated the stability of flavonoid-protein complexes. The observation of oligomeric species on HPLC confirms that flavonoid-induced oligomers are more stable and thus different from *α*-synuclein oligomers obtained without flavonoids that easily dissociate and are not detected by HPLC.

 Interestingly, Figure 8 also shows that the distribution of oligomeric forms and monomers varied depending on the nature of flavonoid. For example, eriodyctiol mostly stabilized dimers and monomers, G-500 mostly monomers, and baicalein mostly oligomers and monomers. 

**Figure 9:**
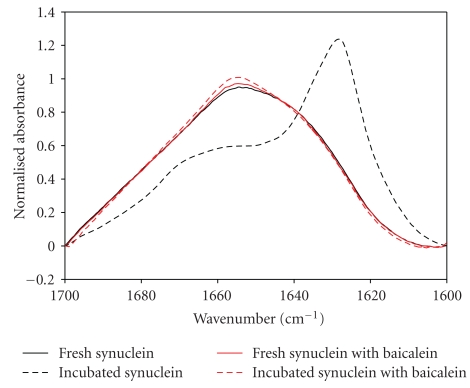
Secondary structure analyses of *α*-synuclein in the presence of baicalein by ATR-FTIR (red lines). The spectra of *α*-synuclein alone before and after incubation was presented for comparison (black lines).

**Figure 10:**
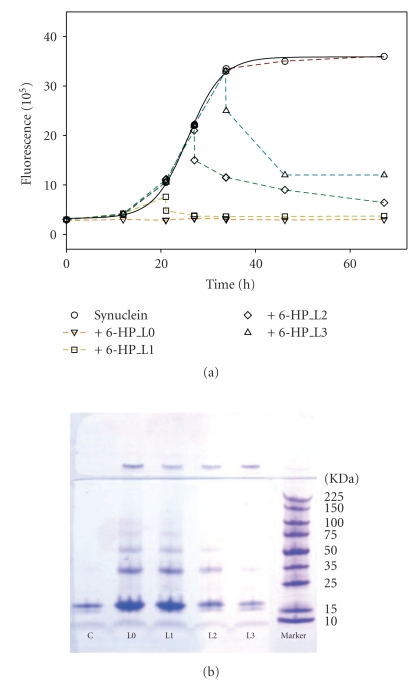
The effect of baicalein on *α*-synuclein fibrillation at different stages by monitoring the kinetics of *α*-synuclein fibrillation (a) and the SDS-PAGE of the supernatants at the end of reaction (b) for the sample with baicalein added at various times, that is, 0 hour (sample L0), 12 hours (end of nucleation, sample L1), 21 hours (middle of elongation, sample L2), and 33 hours (end of elongation, sample L3).

All inhibitory flavonoids affected *α*-synuclein aggregation and lead to the inhibition of fibril formation, accumulation of oligomeric species, and stabilization of monomers and oligomers. Therefore, at the next stage, we analyzed the conformational properties of flavonoid-stabilized *α*-synuclein species. To this end, attenuated total reflectance FTIR was applied to detect the secondary structure of flavonoid-stabilized soluble species of *α*-synuclein. The FTIR spectrum of fresh *α*-synuclein with baicalein exhibits a typical unfolded structure, similar to fresh *α*-synuclein alone, with the peak centered at 1652 cm^−1^ (Figure 9, solid lines). After 3 days of incubation, the spectrum of the protein alone changed into a structure with significant *β*-sheet, with the peak at 1630 cm^−1^, indicative of fibril formation (Figure 9, black lines), whereas the spectrum of the protein with the compound did not exhibit significant structural changes (Figure 10, red lines), reflecting that baicalein inhibits *α*-synuclein fibrillation by stabilizing the natively unfolded conformation of the protein.

### 3.3. Analysis of Flavonoid Inhibitory Effects on Various Stages of Fibrillogenesis

The inhibition of *α*-synuclein fibrillogenesis by flavonoids may involve two different events, the inhibition of nucleus formation occurring during lag time or the inhibition of fibril elongation corresponding to the linear increase in the ThT fluorescence intensity. To resolve it, one of the strong inhibitory flavonoids, 6-HP, was added at various times during incubation of *α*-synuclein. As shown on Figure 10(a), 6-HP stopped the progress of fibrillation regardless of the time it was added, namely, at 0 hour (sample L0), 12 hours (end of nucleation, sample L1), 21 hours (middle of elongation, sample L2), and 33 hours (end of elongation, sample L3). Addition of 6-HP at any time during nucleation resulted in the complete inhibition of fibril formation. This indicated that 6-HP could act on the late steps of nucleation such as formation of oligomers. When 6-HP was added during linear growth of fibrils or at the end of fibrillation, the decrease in ThT intensity and disaggregation of formed fibrils were observed. The initial drop was mostly due to the quenching of ThT fluorescence by the flavonoid, and the subsequent decrease in ThT signal was due to disaggregation of the existing fibrils. This was confirmed by EM and SDS-PAGE. The rates of disaggregation were similar and did not depend on time of flavonoid addition. It was consistent with explanation that the fibrillar species were similar in structure and mechanism of disaggregation was the same.

SDS-PAGE of *α*-synuclein supernatant fractions with 6-HP added at various times of incubation was run to verify the results of ThT assays (Figure 10(b)). SDS-PAGE analysis demonstrated the considerable build-up of monomers, dimers, trimers, and higher MW oligomers as long as the addition of 6-HP was prior to nucleation (samples L0, L1). This indicated that the most efficient inhibition took place during, or prior to, nucleation. When 6-HP was added after the nucleation in the middle of or at the end of fibril elongation (L2 and L3), less amount of monomers and oligomers was detectable. 

Similar results were observed for *α*-synuclein with other strong inhibitory flavonoids such as baicalein, EGCG, and T-415. It can be concluded that the inhibitory flavonoids stopped the progress of fibrillation at any stage. Importantly, the addition of flavonoids to the preformed fibrils resulted in almost complete fibril disaggregation. However, the most efficient inhibition of *α*-synuclein fibrillation by the flavonoids took place on or prior to nucleation through formation of the flavonoids-stabilized monomeric and oligomeric forms of the protein.

### 3.4. Structural Features of *α*-Synuclein Fibrillation Inhibitors

Our results showed that a variety of flavonoids can inhibit the fibril formation of *α*-synuclein. Some flavonoids can also disaggregate existing fibrils. Among the 48 flavonoids tested, several compounds exhibited stronger inhibitory effects than others despite the overall structural similarity of the flavanoid family. The aim of this study was to eventually elucidate the relationship between the molecular structure of a series of structurally related flavonoids and their ability to inhibit *α*-synuclein fibrillation.

The molecular structural requirements that appear necessary to provide a flavonoid the ability to inhibit *α*-synuclein fibrillation were determined to be a vicinal dihydroxyphenyl moiety. In another words, flavonoids without any vicinal dihydroxyphenyl moieties have no inhibitory effect on *α*-synuclein fibrillation. 

**Figure 11:**
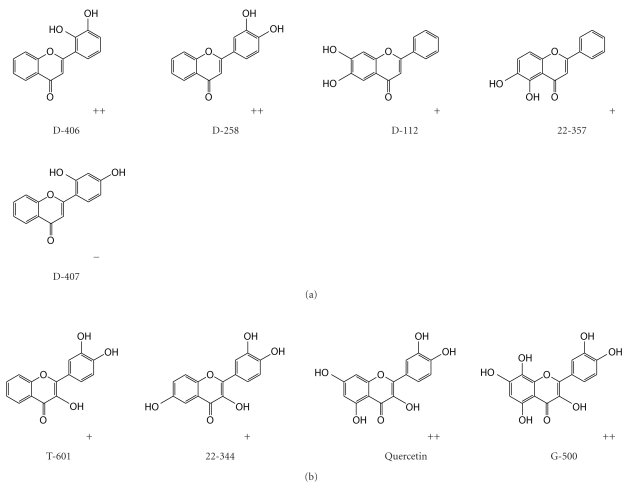
Structural comparisons among the 5 flavones with two hydroxyl groups (a). Structural comparisons among the 4 flavonols with different numbers of individual hydroxyl groups or dihydroxyl groups (b).

The substitution pattern, that is, the ring to locate in and the position on the ring, of the dihydroxyl groups could also affect the inhibitory activity but not necessarily. For example, comparisons were made among the following 5 flavones (Figure 11(a)). The 2 flavones, D-258 with the 3′,4′-dihyhroxy substitution pattern on ring B, and D-406 with the 2′,3′-dihyhroxyl group in ring B, have a similarly good inhibitory effect on *α*-synuclein fibrillation. Rather than the o-dihydroxy structure in the B ring, the other 2 flavones, D-112 and 22-357, have hydroxyl substituents in a catechol structure on the A ring at different positions. The 2 compounds also have a similar inhibitory effect on *α*-synuclein fibrillation, but weaker than the effect from D-258 and D-406. In contrast to the above 4 flavones, D-407 does not have a vicinal dihydroxyphenyl moiety, nor does it have any inhibitory effect on *α*-synuclein fibrillation (see activity in Supplementary Table S1). 

Moreover, the difference in the number of vicinal dihydroxyl substituents and the number of individual hydroxyl groups also brings some difference in the inhibitory activities of flavonoids. Generally, the larger the number, the stronger it is. For instance, the 4 flavonols, T-601, 22-344, quercetin, and G-500, all of them have the 3′,4′- dihyhroxy substitution pattern on ring B, (Figure 11(b)) conferring them the inhibitory activity. However, T-601 does not have any individual hydroxyl group in the A ring, 22-344 has one single hydroxyl group in ring A, quercetin has 2 individual hydroxyl groups in ring A, and G-500 has 3 hydroxyl groups with 2 of them adjacent. Not surprisingly, the inhibitory activity is in the increasing order of T-601 < 22-344 < quercetin < G-500 (data not shown). 

**Figure 12:**
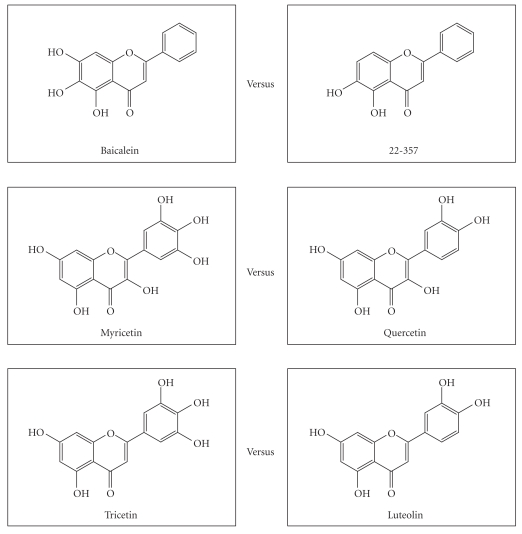
Flavonoids with three vicinal hydroxyl groups exhibit enhanced inhibitory effects on *α*-synuclein fibrillation. Comparisons are made between the three pairs of flavonoids.

**Figure 13:**
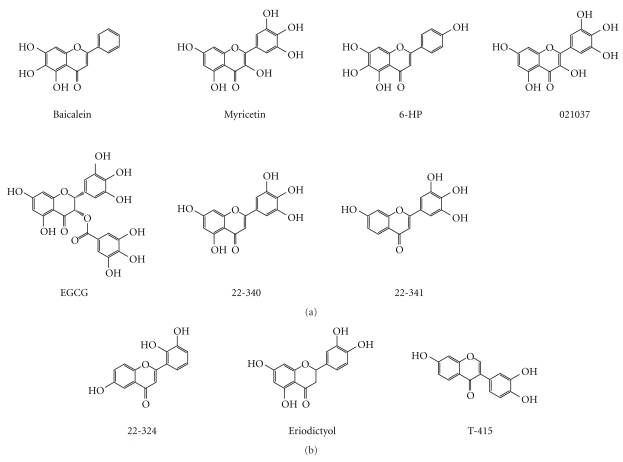
Structures of the strong inhibitory flavonoids of *α*-synuclein fibrillation, with three (a) and two (b) vicinal hydroxyl groups.

Flavonoids with three vicinal hydroxyl groups exhibited the enhanced inhibitory effects on *α*-synuclein fibrillation (structures in Figure 12). For example, 6-HP exhibited stronger inhibitory activity than 22-357 (Figure 2); myricetin was stronger than quercetin, and tricetin was stronger than luteolin (data not shown). Interestingly, all the flavonoids (except 021037) with three vicinal hydroxyl groups from our screening were found to be strong inhibitors, that is, completely inhibited the fibril formation within 3 days of incubation (Figure 13(a)). The essentially weaker inhibitory effects of 021037 could result from the lack of 2,3-double bond in this flavonoid. On the other hand, only three flavonoids without three vicinal hydroxyl groups from this screening were found to exhibit strong inhibitory effect on *α*-synuclein fibrillation. They were as follows: T-415 an isoflavonoid, eriodictyol a flavanone, and 22-324 a flavone (Figure 13(b)). 

**Figure 14:**
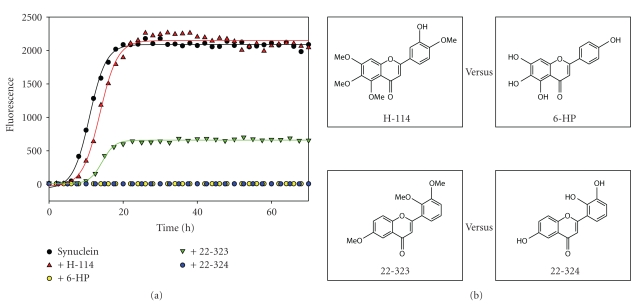
Loss of inhibitory effects of flavonoids on *α*-synuclein fibrillation with hydroxyl groups replaced by methoxyl groups. Comparison is made between 6-HP versus H-114, and 22-324 versus 22-323.

Inversely, the flavonoids with structures exactly the same or very similar (the number and position of ring substitutions) to those of strong inhibitors, but with hydroxyl groups replaced by methoxyl groups, did not have any inhibitory effects on *α*-synuclein fibrillation, as exemplified by the following pairs of flavonoids H-114 versus 6-HP and 22-324 versus 22-323 (Figure 14). The details of the molecular mechanisms underlying the flavanoid-induced inhibition of *α*-synuclein fibrillation were analyzed recently [68]. It was shown that the noncovalent binding of the inhibitory flavonoids to *α*-synuclein and the covalent modification by the flavonoid quinone led to the restriction of the conformational changes in this natively unfolded protein and to the stabilization of soluble flavonoid-modified species of *α*-synuclein (monomers and oligomers). All of these factors rather than a single one contribute to the inhibition of *α*-synuclein fibrillation induced by the flavonoid. The structural requirements for flavonoid to be a good inhibitor of *α*-synuclein fibrillation were vicinal dihydroxyphenyl moieties, irrespectively of their positions in the rings. Flavonoids with three vicinal hydroxyl groups exhibited enhanced inhibitory effects on *α*-synuclein fibrillation. The antioxidant activities of flavonoids were generally correlated with their in vitro inhibitory effects on *α*-synuclein fibrillation [68]. 

### 3.5. Implications for the Treatment of PD

PD has a multifactorial etiology depending on genetic susceptibility and environmental factors. From epidemiological and environmental studies, oxidative stress, transition metal accumulation, and inflammation appear to play a role in the pathology of PD. However, the detailed molecular mechanisms of triggering the oxidative stress as well as targets of transition metal action are not known. The cause of inflammation is also unknown. From genetic etiological studies, *α*-synuclein misfolding and aggregation as well as impairment in the cellular folding machinery appear to be important in the early onset of PD. However, functions of *α*-synuclein, a cause of protein aggregation, and its role in the triggering of neurodegeneration are still unknown. Recently, it has been proposed that *α*-synuclein may interact with mitochondrial membranes and affects their structures. This interesting idea may consolidate genetic and environmental studies of PD etiology. *α*-Synuclein could be a key factor, upon which oxidative stress or transition metals act resulting in protein aggregation or oligomerization that in turn affect mitochondrial membranes leading to mitochondrial dysfunctions including elevated ROS production, leakage of cellular membrane compartments, cell death, and inflammation. Therefore, the prevention of protein aggregation and oligomerization is an attractive strategy in combating the neurodegeneration because it acts on the very beginning of the proposed cellular pathway leading to cell death. In our study, we analyzed 48 flavonoids for their efficiency to inhibit *α*-synuclein aggregation in vitro by stabilizing nonpathogenic protein conformations. Majority of the flavonoids inhibit *α*-synuclein polymerization either delaying or completely abolishing fibril formation. The thermodynamic distributions of *α*-synuclein species are tremendously altered in the presence of strong flavonoid inhibitors. Flavonoids such as 6-HP, eriodictyol, G-500, and baicalein tightly bind to the protein and greatly stabilize its natively unfolded conformation. The major species existing in the thermodynamic equilibrium in the presence of flavonoids are oligomers and monomers. Flavonoid compounds show different ratios in distribution of monomers and oligomers indicating that they stabilize mostly either monomeric or oligomeric conformations. Most importantly, strong inhibitors disaggregate preformed fibrils. 

Substantial evidence reveals that flavonoids act in vivo as health-beneficial and neuroprotective agents. For example, baicalein, a typical flavonoid compound, is the main component of a traditional Chinese herbal medicine Scutellaria baicalensis. Recent studies have shown that baicalein protects rat cortical neurons from amyloid *β*-induced toxicity by its inhibition of lipoxygenase [69]. Natural medicines containing these compounds have been reported to have beneficial effects in treating memory loss and dementia [70, 71]. Another flavonoid, (−)-epigallocatechin-3-gallate (EGCG), has been shown neuroprotective in an MPTP-induced animal model of dopaminergic degeneration [64]. Their data from the yeast model also demonstrated that flavonoids counteract the cytotoxic properties of *α*-synuclein toxicity directly in a cellular context [65]. Epidemiological studies suggest an inverse relation between flavonoid intake and incidence of chronic diseases, such as coronary heart disease [72–74], as well as dementia and other neurodegenerative diseases [63, 70].

However, little is currently known about the mechanism of flavonoid action in vivo. There are two models that explain the flavonoid health beneficial effects. First model suggests that the antioxidant properties of flavonoids are important in combating oxidative stress by scavenging radicals and by metal chelation. However, even with very high flavonoid intakes, plasma and intracellular flavonoid concentrations in humans are likely to be 100–1,000 times lower than concentrations of other antioxidants, such as ascorbate or glutathione. Furthermore, most circulating flavonoids are actually flavonoid metabolites, some of which have lower antioxidant activity than the parent flavonoid. For these reasons, the relative contribution of dietary flavonoids to plasma and tissue antioxidant function is most likely to be relatively minor [75, 76]. 

The second model postulates that many of the biological effects of flavonoids can be related to their ability to modulate cell-signaling pathways rather than to act as antioxidants. For instance, several signaling mechanisms were suggested to explain polyphenol-induced protection against amyloid cytotoxicity [77] and the neuroprotective effects of flavonoids [78]. The validity of the hypothesis is supported by the facts that intracellular concentrations of flavonoids required to affect cell signaling pathways are considerably lower than those required to impact cellular antioxidant capacity, and flavonoid metabolites may still retain their ability to interact with cell signaling proteins, even if their antioxidant activity is diminished.

Our results have demonstrated that flavonoids significantly affect *α*-synuclein propensity to aggregate in vitro through binding and stabilizing the certain protein conformations. This direct act of flavonoids on protein aggregation is a highly possible mechanism of flavonoid action in vivo. The apparent  *K*_*d*_ of flavonoid-*α*-synuclein complexes is in range of 200–800 nm, which is sufficient for binding of flavonoids in vivo [68]. Furthermore, there is a certain correlation between antioxidant activities of flavonoids and their in vitro inhibitory effects on *α*-synuclein fibrillation; thus it is possible that the antioxidant potential of flavonoids may relate not only to the scavenging of radicals and metals but also to binding to amyloidogenic proteins such as *α*-synuclein, locking the certain protein structures, and abolishing protein aggregation. 

It has been suggested that the precursors of fibrils, the transient oligomers, and protofibrils on the aggregation pathway in neurodegenerative diseases might be more toxic than fibrils [21, 22]. Hence, it can be argued that the flavonoid-induced oligomers from our study could be more cytotoxic than fibrils. Several studies have been done to test the cytotoxicity of one of the flavonoid-synuclein complexes, the baicalein-stabilized oligomers, and have demonstrated their benign effects on membrane permeability [38], cell culture toxicity [79], and neuron degeneration in worms and mice. Masuda et al. have reported that, unlike *α*-synuclein fibrils and protofibrils, flavonoid-induced soluble oligomeric species did not reduce the viability of human dopaminergic neuroblastoma SH-SY5Y cells [79]. These findings suggest that the soluble oligomers formed in the presence of inhibitory flavonoids may not be toxic to human neurons and that these flavonoids may therefore have therapeutic potential for PD and other amyloidoses. 

Flavonoids are among the most secure and reliable substances available now for preventing various diseases including maladies related to the oxidative-stress and neurodegeneration. In this study, we have demonstrated that flavonoids can directly act on aggregation of *α*-synuclein, being able to inhibit fibrillation and disaggregate the preformed fibrils into monomers and nonpathogenic oligomers. This implies that flavonoids may have been already combating PD, and that diets rich in flavonoids may be effective in preventing and curing the disorder.

##  Supporting Information Available

Experimental details of the inhibitory activities of each flavonoid analyzed in this study are available; the figures illustrate instant changes in the intensity of ThT fluorescence upon adding 50 *μ*M flavonoids directly to the existing *α*-synuclein fibril solution. This material is available free of charge via the Internet at http://pubs.acs.org.

## Supplementary Material

Supplementary materials represent experimental details of the inhibitory activities of each flavonoid analyzed in this study (Table S1). Data presented in Figure S1 illustrate instant changes in the intensity of ThT fluorescence upon adding 50 *μ*M flavonoids directly to the solution of existing *α*-synuclein fibrils.

## Abbreviations

ThT: Thioflavin T
DMSO: Dimethyl sulfoxide
ATR-FTIR: Attenuated total reflectance fourier transform infrared spectroscopy
UV: Ultra violet
CD: Circular dichroism
SDS-PAGE: Sodium dodecylsulfate polyacrylamide gel electrophoresis
IEF: Isoelectric focusing
DMPO: 5,5-Dimethyl-1-Pyrroline-N-Oxide
ESI-MS: Electrospray ionization mass spectrometry
PD: Parkinson's disease.
