# Supplementary material for: Effects of Various Flavonoids on the α-Synuclein Fibrillation Process
Source: Parkinsons Dis. 2010 Jan 28;2010:650794. doi: 10.4061/2010/650794 (PMC2957276; doi:10.4061/2010/650794)

Supplementary Materials

Table S1. Classification of inhibitors of α-synuclein fibrillation

- Non-inhibitors.
+ Weak inhibitors, with a lag time 2 or 3 times longer than control;
++ Good inhibitory effect; with a lag time 4 or 5 times longer than control;
+++ Excellent inhibitors, completely inhibit synuclein fibril formation after 3 days incubation;

Flavonols:

Catalog NO. / Name Synonym	Activity	8	7	6	5	3	2'	3'	4'	5'	6'
Quercetin<br>3,5,7,3',4'-Pentahydroxyflavone dihydrate	++	H	OH	H	OH	OH	H	OH	OH	H	H
Fisetin<br>3,7,3',4'-Tetrahydroxyflavone	+	H	OH	H	H	OH	H	OH	OH	H	H
T-601<br>3',4'-Dihydroxyflavonol	+	H	H	H	H	OH	H	OH	OH	H	H
22-344<br>3,6,3',4'-Tetrahydroxyflavone	+	H	H	OH	H	OH	H	OH	OH	H	H
22-318<br>3,6,2',3'- Tetrahydroxyflavone	+	H	H	OH	H	OH	OH	OH	H	H	H
G-500 / Gossypetin<br>3,5,7,8,3',4'-Hexahydroxyflavone	++	OH	OH	H	OH	OH	H	OH	OH	H	H
C-101 / Myricetin<br>3,5,7,3',4',5'-Hexahydroxyflavone,	+++	H	OH	H	OH	OH	H	OH	OH	OH	H
Rutin<br>Quercetin-3-rutinoside	+	H	OH	H	OH	Rut	H	OH	OH	OH	H

Catalog NO. / Name/ Synonym	Activity	8	7	6	5	3	2'	3'	4'	5'	6'
K-102 / Kaempferol<br>3,5,7,4'-Tetrahydroxyflavone	-	H	OH	H	OH	OH	H	H	OH	H	H
020065 / Isorhamnetin<br>3'-Methoxy-3,5,7,4'-Tetrahydroxyflavone	-	H	OH	H	OH	OH	H	H	OH	OMe	H
020067 / Galangin<br>3,5,7-Trihydroxyflavone	-	H	OH	H	OH	OH	H	H	H	H	H
021140S / Tamarixetin<br>4'-Methoxy-3,5,7,3'-Tetrahydroflavone	-	H	OH	H	OH	OH	H	OH	OMe	H	H

Flavones:

Catalog NO. / Name<br>Synonym	Activity	8	7	6	5	3	2’	3’	4’	5’	6’
22-324<br>6,2',3'- Trihydroxyflavone	+++	H	H	H	H	H	OH	OH	H	H	OH
D-406<br>2',3'- Dihydroxyflavone	++	H	H	H	H	H	OH	OH	H	H	H
D-258<br>3',4'- Dihydroxyflavone	++	H	H	H	H	H	H	OH	OH	H	H
D-116<br>5,6-Dihydroxy-7-Methoxyflavone	++	H	OMe	OH	OH	H	H	H	H	H	H
22-357<br>5,6- Dihydroxyflavone	+	H	H	OH	OH	H	H	H	H	H	H
22-336<br>6,7,3'- Trihydroxyflavone	+	H	OH	OH	H	H	H	OH	H	H	H
D-112<br>6,7- Dihydroxyflavone	+	H	OH	OH	H	H	H	H	H	H	H
Luteolin<br>5,7,3',4'-Tetrahydroxyflavone	+	H	OH	H	OH	H	H	OH	OH	H	H
22-340 / Tricetin<br>5,7,3',4',5'-Pentahydroxyflavone	+++	H	OH	H	OH	H	H	OH	OH	OH	H
22-341<br>7,3',4',5'- Tetrahydroxyflavone	+++	H	OH	H	H	H	H	OH	OH	OH	H
021165 / 6-HP<br>5,6,7,4'-Tetrahydroxyflavone	**+++**	H	OH	OH	OH	H	H	H	OH	H	H
B-101 / Baicalein<br>5,6,7- Trihydroxyflavone	**+++**	H	OH	OH	OH	H	H	H	H	H	H

Catalog NO. / Name<br>Synonym	Activity	8	7	6	5	3	2’	3’	4’	5’	6’
22-323<br>6,2',3'-<br>Trimethoxyflavone	**-**	H	H	H	H	H	OMe	OMe	H	H	OMe
D-407<br>2',4'-<br>Dihydroxyflavone	**-**	H	H	H	H	H	OH	H	OH	H	H
021104S /<br>Chrysoeriol<br>4',5,7-Trihydroxy-3'-<br>Methoxyflavone	**-**	H	OH	H	OH	H	H	H	OH	OMe	H
021108S / Diosmetin<br>5,7,3'-Trihydroxy-4'-<br>Methoxyflavone	**-**	H	OH	H	OH	H	H	OH	OMe	H	H
Wogonin<br>5,7-Dihydroxy-8-<br>Methoxyflavone	**-**	OMe	OH	H	OH	H	H	H	H	H	H
H-114<br>3'-Hydroxy-5,6,7,4'-<br>Tetramethoxyflavone	**-**	H	OMe	OMe	OMe	H	H	OH	OMe	H	H

Flavanones:

Catalog NO. / Name<br>Synonym	Activity	8	7	6	5	3	2’	3’	4’	5’	6’
020056 / Eriodictyol<br>2-[3,4-Dihydroxyphenyl] 2,3-dihydro-5,7-dihydroxy-4H-1benzopyran-4-one	+++	H	OH	H	OH	H	H	OH	OH	H	H

Catalog NO. / Name<br>Synonym	Activity	8	7	6	5	3	2’	3’	4’	5’	6’
H-103 / Hesperetin<br>5,7,3'-Trihydroxy-4'-methoxyflavanone	-	H	OH	H	OH	H	H	OH	OMe	H	H
020091 / Homoeriodictyol<br>5,7,4'-Trihydroxy-5'-methoxyflavanone	-	H	OH	H	OH	H	H	H	OH	OMe	H
Hesperidin/<br>Hesperetin-7-O-rutinoside	-	H	Rut	H	OH	H	H	OH	OMe	H	H

Flavanols:

Catalog NO. / Name<br>Synonym	Activity	8	7	6	5	3	2’	3’	4’	5’	6’
D-105 / Fustin<br>3,7,3',4'- Tetrahydroxyflavone	+	H	OH	H	H	OH	H	OH	OH	H	H
021037<br>3,3',4',5',7-Pentahydroxyflavanone	+	H	OH	H	H	OH	H	OH	OH	OH	H

Non-inhibitors were not tested.

Isoflavonoids:

Catalog NO. / Name<br>Synonym	Activity	8	7	6	5	2	2’	3’	4’	5’	6’
T-116<br>6,7,4'- Trihydroxyisoflavone	+	H	OH	OH	H	H	H	H	OH	H	H
T-415<br>7,3',4'- Trihydroxyisoflavone	+++	H	OH	H	H	H	H	OH	OH	H	H

Catalog NO. / Name<br>Synonym	Activity	8	7	6	5	2	2’	3’	4’	5’	6’
19-612<br>3',4'-Dimethoxy-7-hydroxyisoflavone	-	H	OH	H	H	H	H	OMe	OMe	H	H
D-101 / Daidzein<br>7,4'-Dihydroxyisoflavone	-	H	OH	H	H	H	H	H	OH	H	H
F-103 / Formononetin<br>7-Hydroxy-4'-methoxyisoflavone	-	H	OH	H	H	H	H	H	OMe	H	H
Biochanin A<br>5,7-Dihydroxy-4'-methoxyisoflavone	-	H	OH	H	OH	H	H	H	OMe	H	H

Catechins:

Catalog NO. / Name<br>Synonym	Activity	8	7	6	5	4	3	2’	3’	4’	5’	6’
020976S / Catechin<br>(+)-3,3'4',5,7-Flavanepentol<br>(2H)-benzopyran-3,5,7-triol /<br>(2R,3R)-2-(3,4-Dihydroxyphenyl)-<br>3,4-dihydro-1	+	H	OH	H	OH	H	OH	H	OH	OH	H	H
Epicatechin gallate (ECG)<br>((2R,3R) -2-(3,4-Dihydroxyphenyl)<br>-3,4-dihydro-1<br>(2H) -benzopyran-3,5,7-triol 3-<br>(3,4,5-trihydroxybenzoate)	+	H	OH	H	OH	H	gallate	H	OH	OH	H	H
EpigalloCatechin Gallate<br>(EGCG)<br>(2R,3R)-2-(3,4,5-<br>Trihydroxyphenyl)-3,4-dihydro-<br>1(2H)-benzopyran-3,5,7-triol 3-<br>(3,4,5-trihydroxybenzoate)	+++	H	OH	H	OH	H	gallate	OH	OH	OH	H	H

Non-inhibitors were not tested.

Anthraquinones:

Catalog NO. / Name<br>Synonym	Activity	8	7	6	5	3	2’	3’	4’	5’	6’
020411 / Alizarin<br>1,2-Dihydroxyanthraquinone	+										

Catalog NO. / Name<br>Synonym	Activity	8	7	6	5	3	2’	3’	4’	5’	6’
Chrysophanol<br>1,8-Dihydroxy-3-methylanthraquinone	-										
Emodin<br>1,3,8-Trihydroxy-6-methylanthraquinone	-										

Figure S1. Instant changes in the intensity of ThT fluorescence upon adding 50 μM flavonoids directly to the existing α-synuclein fibril solution

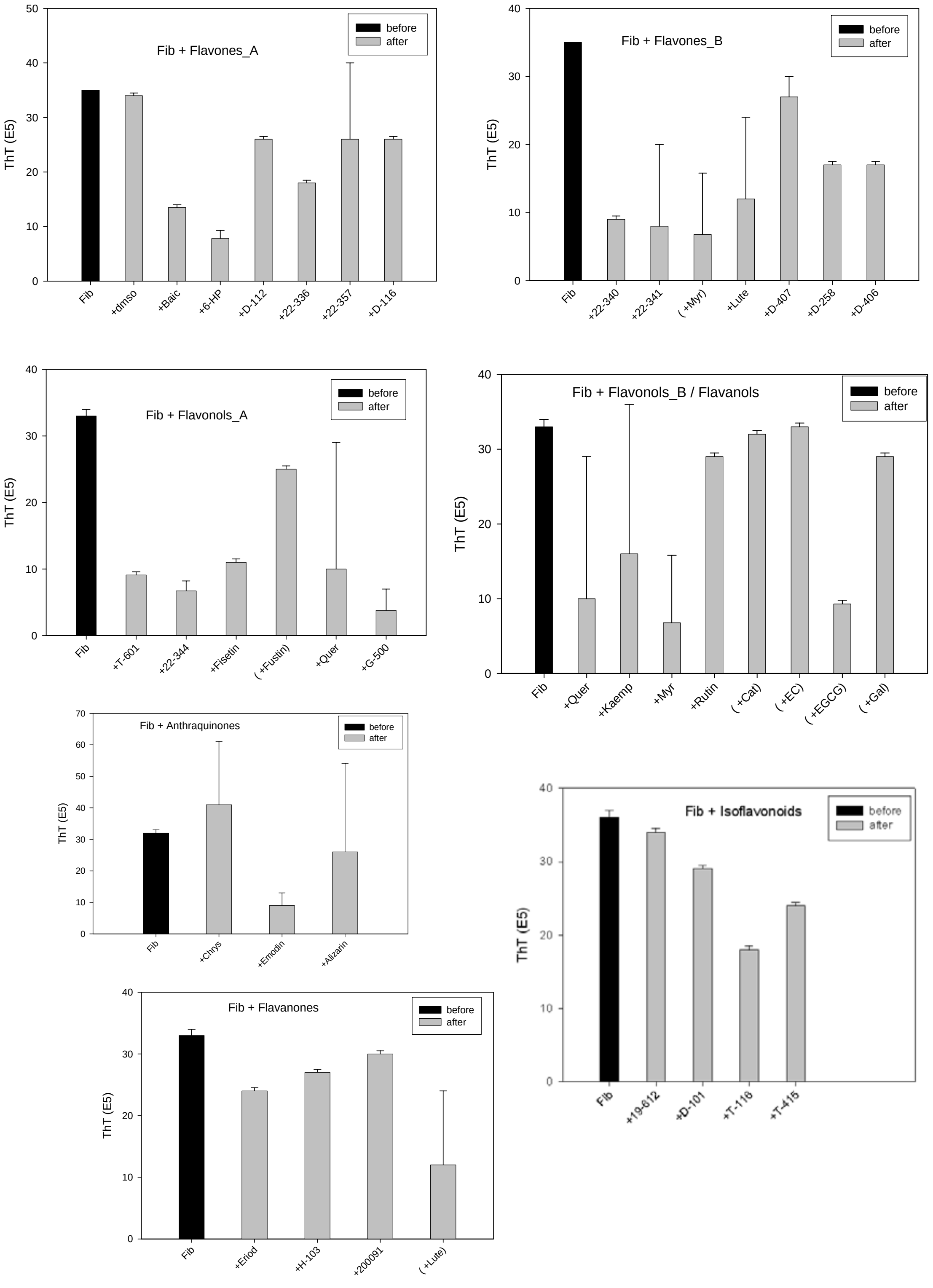

Supplement: Supplementary file 1 — Supplementary materials represent experimental details of the inhibitory activities of each flavonoid analyzed in this study (Table S1). Data presented in Figure S1 illustrate instant changes in the intensity of ThT fluorescence upon adding 50 μM flavonoids directly to the solution of existing α-synuclein fibrils. [file 650794.f1.pdf]
